# Antidepressant and Related Neurobiological and Neurophysiological Effects of Add-On Transcranial Direct Current Stimulation in Major Depressive Disorder with Residual Symptoms: A Randomized, Double-Blind Clinical Trial Protocol

**DOI:** 10.3390/mps8050117

**Published:** 2025-10-02

**Authors:** Carmen Concerto, Fabrizio Bella, Cecilia Chiarenza, Alessandro Rodolico, Antonio Di Francesco, Alessia Ciancio, Stefania Lanzafame, Riccardo Spigarelli, Ludovico Mineo, Antonino Petralia, Raffaele Ferri, Massimo Libra, Rita Bella, Manuela Pennisi, Giuseppe Lanza, Maria Salvina Signorelli

**Affiliations:** 1 Psychiatry Unit, Department of Clinical and Experimental Medicine, University of Catania, 95123 Catania, Italy; faber.bella92@gmail.com (F.B.); cecilia.chiarenza11@gmail.com (C.C.); alessandro.rodolico@tum.de (A.R.); antonio.di.francesco96@gmail.com (A.D.F.); alessia.ciancio@gmail.com (A.C.); stefanialanzfame@gmail.com (S.L.); riccardo.spigarelli91@gmail.com (R.S.); ludwig.mineo@gmail.com (L.M.); petralia@unict.it (A.P.); maria.signorelli@unict.it (M.S.S.); 2 Department of Psychiatry and Psychotherapy, Technical University of Munich, TUM School of Medicine and Health, Klinikum Rechts der Isar, 81675 Munich, Germany; 3 Oasi Research Institute-IRCCS, 94018 Troina, Italy; rferri@oasi.en.it (R.F.); giuseppe.lanza1@unict.it (G.L.); 4 Department of Biomedical and Biotechnological Sciences, University of Catania, 95123 Catania, Italy; mlibra@unict.it (M.L.); manuela.pennisi@unict.it (M.P.); 5 Department of Medical, Surgical Sciences and Advanced Technolgies, University of Catania, 95123 Catania, Italy; rbella@unict.it; 6 Department of General Surgery and Medical-Surgical Specialties, University of Catania, 95123 Catania, Italy

**Keywords:** major depressive disorder, transcranial direct current stimulation, neuromodulation, neuroplasticity, inflammation, outcome measures

## Abstract

Major depressive disorder (MDD) is a prevalent and disabling condition. Transcranial direct current stimulation (tDCS) may improve symptoms by modulating neuroplastic and inflammatory mechanisms. This randomized, double-blind, placebo-controlled trial will recruit adult outpatients with MDD showing residual symptoms despite at least four weeks of stable SSRI treatment. Participants will be randomized to active or sham add-on tDCS while continuing their antidepressant regimen. The intervention will consist of 15 sessions over 3 weeks, targeting the left dorsolateral prefrontal cortex (anode F3, cathode F4) at 2 mA for 30 min per session. The primary outcome is the reduction of depressive symptoms measured by the Hamilton Depression Rating Scale-17 (HDRS), with remission defined as HDRS-17 ≤ 7. Secondary outcomes include cognitive performance (attention, executive functioning, memory), serum biomarkers (BDNF, VEGF, NGF, NRG1, angiogenin, IGF1, IL-6, TNF-α), cortical excitability assessed by transcranial magnetic stimulation (motor threshold, silent period, intracortical inhibition/facilitation), and cerebral hemodynamics by transcranial Doppler sonography (blood flow velocity, pulsatility, resistivity). Assessments will occur at baseline, post-treatment, and 3- and 6-month follow-ups. This trial aims to evaluate the efficacy of adjunctive tDCS in MDD with residual symptoms and its biological correlates, bridging clinical improvement with electrophysiological and neurovascular mechanisms.

## 1. Introduction

### 1.1. Background

Major depressive disorder (MDD) is a highly prevalent and debilitating neuropsychiatric condition associated with elevated suicide rates and significant social and economic burden. Globally, it is estimated that over 280 million people of all ages suffer from depression [1], making it one of the leading causes of disability worldwide. The high lifetime prevalence of MDD, combined with its recurrent nature, often complicates treatment outcomes and contributes to the limited effectiveness of conventional therapies [2,3,4,5]. In the context of antidepressant therapy, residual symptoms remain a major clinical concern, affecting a large proportion of patients, even after an initial therapeutic response. These persistent symptoms—including anhedonia, cognitive impairment, insomnia, and anxiety—are strongly associated with functional impairment, reduced quality of life, and a higher risk of relapse or recurrence. Their persistence highlights the importance of treatment optimization, such as dose adjustment, augmentation strategies, or combination approaches, in order to achieve full remission and long-term recovery [6]. Pharmacological treatments typically begin to show effectiveness within 2 to 4 weeks; however, a longer duration may be necessary to achieve clinically significant improvement [7,8]. A delayed or incomplete response is consequently associated with a greater risk of chronicity [9]. Such chronicity appears to be associated with multiple contributing factors, including a sustained inflammatory state, characterized by a complex interplay among the hypothalamic–pituitary–adrenal (HPA) axis, the immune system, the neurovascular units (NVUs), and disruptions in both neurogenesis and neurotransmission [10,11].

Persistent psychological stress induces low-grade systemic inflammation, which may compromise the integrity of the blood–brain barrier by disrupting the NVU—a functional ensemble of vascular cells, glial cells, and neurons—ultimately promoting central neuroinflammation [12,13]. This dysfunction appears to be driven by hyperactivation of the HPA axis, with subsequent excessive cortisol secretion and glucocorticoid receptor resistance, which perpetuate immune dysregulation [14,15]. Experimental models further indicate that elevated corticosterone disrupts the structural and functional integrity of the NVU and inhibits brain endothelial cell proliferation [16,17].

Vascular dysfunction is also associated with increased brain tissue pulsatility, a biomarker of altered cerebral perfusion and vascular dynamics. This phenomenon may activate microglia via the P2X7–inflammasome axis, sustaining inflammation even in the absence of infection or injury [10,18]. Furthermore, the impaired functionality of NVU allows peripheral cytokines and immune cells to enter the brain, sustaining central neuroinflammation [10]. Previous data have shown that elevated levels of pro-inflammatory cytokines (e.g., IL-1β, IL-6, TNF-α) are consistently found in patients with chronic depression [19,20,21] and correlate with impaired neuroplasticity, reduced hippocampal neurogenesis, and disrupted mood-regulating circuits [11]. Indeed, pro-inflammatory cytokines may divert tryptophan metabolism toward the kynurenine pathway, leading to the production of neurotoxic metabolites such as quinolinic acid and a reduction in serotonin availability [22]. This, in turn, increases glutamatergic transmission, thereby contributing to the impairment of hippocampal neurogenesis [10,11].

In the adult brain, neurogenesis primarily takes place within the subgranular zone of the dentate gyrus in the hippocampus [23]. Here, neural stem cells (NSCs) differentiate into mature neurons via a well-defined sequence, supported by neurotrophic factors (NTs), especially brain-derived neurotrophic factor (BDNF) [24]. Another critical neurotrophin is nerve growth factor (NGF), which promotes neuronal survival and differentiation, supports synaptic plasticity, and facilitates angiogenesis through the mediation of vascular endothelial growth factor (VEGF) [25,26]. Furthermore, both NGF and BDNF induce the expression of VEGF via induction of hypoxia-inducible factor-1α (HIF-1α). This mechanism operates even under normoxic conditions, indicating a hypoxia-independent regulatory role of NTs in neuroplasticity [27]. Other NTs, including insulin-like growth factor 1 (IGF-1), angiogenin (Ang), and neuregulin-1 (NRG1), contribute to the regulation of neuronal growth, synaptic plasticity, glucose metabolism, and protein synthesis within neurons [28,29,30]. NTs, particularly BDNF, emerge as key molecular mediators linking vascular health to neuroplasticity, primarily by preserving the functional integrity of the NVU and facilitating synaptic remodeling [31,32].

In this context, the neurotrophic hypothesis of depression posits that MDD is associated with a loss of neurotrophic support, particularly a decrease in BDNF, which leads to neuronal atrophy, reduced synaptogenesis, impaired neurogenesis, and glial loss in key brain regions, such as the hippocampus and dorsolateral prefrontal cortex (DLPFC) [33]. Chronic stress suppresses BDNF expression and disrupts synaptic plasticity, while antidepressant treatments—including SSRIs and NMDA receptor antagonists like ketamine—reverse these effects by enhancing BDNF signaling, restoring synaptic connections, and stimulating the mTOR pathway, which drives synaptic protein synthesis and rapid synaptogenesis [34,35]. Moreover, this model suggests that recovery from depression entails not only the restoration of neurochemical balance but also structural remodeling of brain circuits implicated in mood regulation [33]. In particular, the DLPFC, which maintains strong connections with the limbic system and the orbitofrontal cortex, plays a pivotal role in emotional processing, mood regulation, and executive functioning [36]. Indeed, neuroimaging studies consistently reveal reduced metabolism and perfusion in the left DLPFC among individuals suffering from MDD [37].

From this perspective, several efforts have been made to identify treatment-responsive patients from non-responders, who are more likely to develop a chronic clinical course [8]. A number of biomarkers have been proposed for this purpose. Activity in brain regions such as the subgenual anterior cingulate cortex (sgACC), insula, and prefrontal cortex has been associated with response to various antidepressants and psychotherapies [38]. Regarding blood-based biomarkers, molecules such as C-reactive protein (CRP) and BDNF also appear to hold clinical relevance. Higher CRP levels may predict a better response to noradrenergic antidepressants, whereas lower levels seem to be associated with greater efficacy of SSRIs. Also, early changes in BDNF levels during treatment have been linked to clinical improvement, supporting its role as a state-dependent marker [8].

A growing body of evidence from both human and animal studies indicates that reduced BDNF expression in brain areas involved in emotion regulation—such as the hippocampus and prefrontal cortex—is a hallmark of depression. This decrease is also reflected in peripheral blood levels, supporting BDNF’s potential as a biomarker for both depression severity and treatment response [34]. Antidepressant therapies consistently increase BDNF expression, particularly in hippocampal regions like the dentate gyrus and CA3, with this upregulation closely paralleling clinical improvement [39]. Nevertheless, despite ongoing research into biomarkers for depression, no single marker has yet achieved universal acceptance for diagnosis or predicting treatment response.

To date, clinical evaluation remains the cornerstone of assessment and decision-making in depressive disorders [40]. Non-invasive brain stimulation (NIBS) techniques, including transcranial direct current stimulation (tDCS) and transcranial magnetic stimulation (TMS), have shown therapeutic efficacy in MDD, potentially exerting neuroprotective effects through the upregulation of BDNF and NGF, improved cerebral perfusion, and modulation of neuroinflammatory and oxidative stress pathways [41]. Some evidence suggests an increase in serum BDNF following TMS, supporting a neuroplasticity-based mechanism [42]. However, several other studies, both observational and controlled, failed to detect significant changes in BDNF levels post-treatment, especially in older comorbid subjects, and even among patients who responded clinically [43].

A similar pattern emerged for tDCS. In previous studies, improvements in cognitive domains such as verbal learning, recall, attention, and processing speed were observed following tDCS. Specifically, facilitatory effects of tDCS on synaptic plasticity, mood regulation, and memory seem to be linked to the enhancement of the BDNF/TrkB signaling pathway in the hippocampal CA1 region [44]. Notably, the BDNF Val66Met genotype was found to modulate the effects of tDCS: individuals with the Val/Val variant showed greater gains in verbal memory following high-dose stimulation [45]. Nonetheless, some evidence suggests that tDCS may not consistently affect peripheral BDNF levels, even in the presence of clinical improvements [42]. Nevertheless, since BDNF is not the sole factor involved in neuroplasticity, further clinical trials have been recommended to explore correlations between tDCS outcomes and various neurotrophic and inflammatory markers [46]. In clinical settings, anodal tDCS applied over the left DLPFC seems to significantly reduce depressive symptoms, especially in patients with mild to moderate MDD or in combination with pharmacotherapy [47]. In fact, tDCS delivers a weak electrical current through the scalp, inducing neuronal depolarization under the anode and hyperpolarization under the cathode. This technique mimics synaptic processes like long-term potentiation (LTP) and long-term depression (LTD), with antidepressant effects linked to changes in N-methyl-D-aspartate (NMDA) receptor activity [48]. Specifically, an antidepressant effect of anodic stimulation sessions applied on the left DLPFC at intensities between 1 and 2 mA has been demonstrated [49]. In individuals with vascular depression, these effects are particularly beneficial for improving regional cerebral blood flow and addressing cognitive and executive deficits [50,51], which are often resistant to traditional pharmacological treatments [52].

Regarding safety, there is no evidence of serious adverse effects or significant tissue damage attributable to tDCS [53]. Exceptions include transient skin lesions caused by the electrodes, as well as minor side effects such as itching, tingling, headaches, discomfort, and a burning sensation (reported at rates of 8.7% versus 10%), none of which occurred significantly more frequently than with sham tDCS [54]. The responses to sham tDCS also warrant better characterization. Most studies were randomized controlled trials (RCTs) with sham controls designed to simulate stimulation without affecting clinical presentation, inflammation, or plasticity. Overall, prior findings underscore the need to standardize sham tDCS protocols, as the sham response was notably large [55].

### 1.2. Aim and Hypothesis

(i) This randomized, sham-controlled, double-blind trial primarily aims to evaluate the effects of tDCS as an adjunct to pharmacological treatment in MDD outpatients who continue to experience residual depressive symptoms despite adequate SSRI therapy.
(ii) Secondary outcomes are as follows: to assess the impact of tDCS on cognitive performance; to examine pre- and post-tDCS variations in laboratory, neurophysiological, and neuropsychological parameters, including cortical excitability, hemodynamic measures, and neuroplasticity factors, which may underlie the long-term efficacy of the treatment.

Experimental hypotheses:

(i) The primary hypothesis is that participants receiving active tDCS will demonstrate a significant reduction in residual symptoms as measured by the Hamilton Depression Rating Scale—17 (HDRS-17).
(ii) Secondary hypotheses are as follows: the active tDCS group is expected to show a significant reduction in cognitive symptomatology compared to the sham group, as measured by the Montreal Cognitive Assessment (MoCA), Frontal Assessment Battery (FAB), and Stroop Color–Word Interference Test (Stroop T); to show a significant change in serum concentrations of neurotrophic factors (BDNF, VEGF, Nrg1, Ang, IGF1, NGF) and inflammatory cytokines (IL-6, TNF-α), and a correlation with the persistence or remission of residual depressive symptoms. Furthermore, a correlation is expected between the reduction of residual symptoms following tDCS and changes in cortical excitability parameters, as assessed through TMS measures, as well as between improvements in residual depressive symptoms following tDCS and hemodynamic parameters assessed by transcranial Doppler sonography (TCD).

## 2. Experimental Design

**Figure 1:**
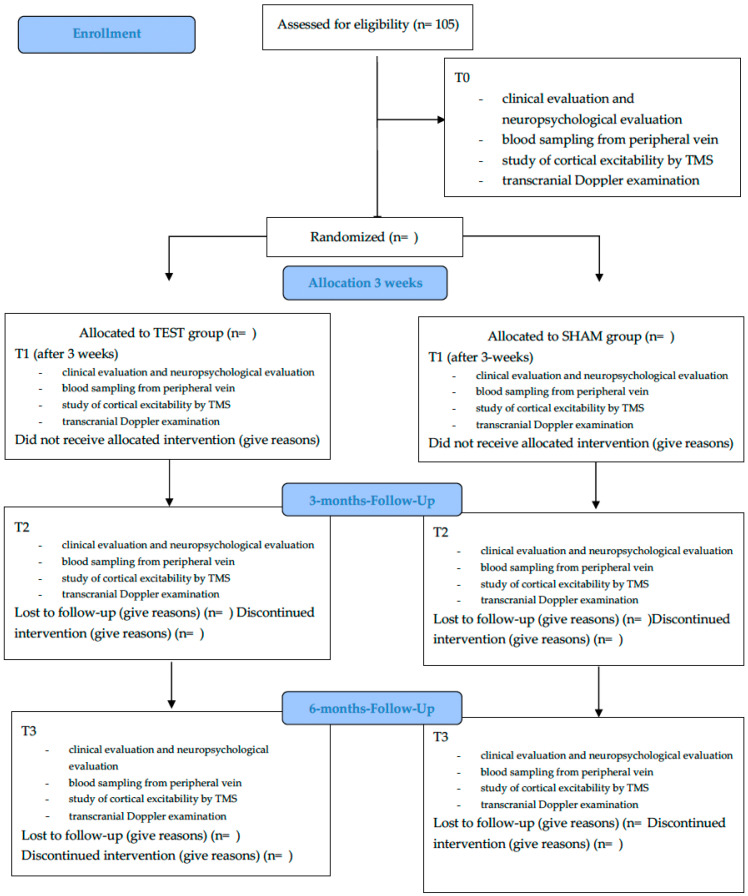
Flow diagram of the study protocol. T0 = baseline; T1 = immediately after the intervention; T2 = 3 months after the intervention; T3 = 6 months after the intervention; TMS = transcranial magnetic stimulation.

The study protocol is a randomized, double-blind clinical trial involving parallel groups, with both patients and evaluators blinded to the treatment allocation. The study will span 24 months and will include clinical follow-ups at three and six months post-treatment. The treatment phase itself will last three weeks. A total of 105 participants are expected to be enrolled. Recruitment will take place at the Psychiatry Unit of the University Hospital Policlinico “G. Rodolico–San Marco” of Catania (Italy). Existing pharmacological therapies for depressive disorders will not be discontinued or substantially altered during the study protocol (Figure 1).

### 2.1. Study Population

The study will enroll outpatients diagnosed with MDD according to the criteria outlined in the DSM-5-TR. Participant recruitment will be conducted at the Psychiatry Unit of the University Hospital Policlinico “G. Rodolico–San Marco” of Catania (Italy). Potential participants will be identified through outpatient services and referred by psychiatrists within the hospital network. Weekly screening meetings will be held to evaluate eligibility based on medical records and direct interviews. Recruitment is expected to take place over a 12-month period.

Eligible participants must meet the following criteria:

(i) Age between 18 and 65 years.
(ii) Residual depressive symptoms despite at least 4 weeks of stable SSRI treatment at an antidepressant dosage, described as follows: fluoxetine 20 mg/day; sertraline 100 mg/day; paroxetine 20 mg/day; citalopram 20 mg/day; escitalopram 10 mg/day; fluvoxamine 100 mg/day. The approximate equivalent dose for each molecule (often referenced to fluoxetine 20 mg as a standard unit) [56] is as follows: fluoxetine 20 mg; sertraline 50 mg; paroxetine 20 mg; citalopram 20 mg; escitalopram 10 mg; fluvoxamine 100 mg.
(iii) A Hamilton Depression Rating Scale–17 (HDRS-17) score > 7 at screening. The HDRS-17 cut-off was selected because a score ≤ 7 is conventionally regarded as remission, whereas scores above this threshold indicate the persistence of residual symptoms. The use of HDRS-17, therefore, provides a standardized and widely accepted method to capture patients who, although they may have improved with treatment, continue to experience a clinically meaningful symptom burden that prevents full remission.
(iv) Capacity to provide written informed consent and comply with all study procedures.

Exclusion criteria are as follows:

(i) Cognitive impairment: Mini-Mental State Examination (MMSE) < 18 or Clinical Dementia Rating (CDR) > 2;
(ii) Neurological disorders: history of stroke, multiple sclerosis, major head injury, epilepsy, or other clinically significant neurological conditions;
(iii) Psychiatric comorbidity: diagnosis of schizophrenia, schizoaffective disorder, bipolar disorder, other primary psychiatric conditions, and personality disorders (excluding comorbid anxiety disorders if secondary to MDD);
(iv) Severe medical conditions: acute or chronic illnesses not adequately controlled (e.g., uncontrolled hypertension, diabetes, or systemic diseases);
(v) Endocrine disorders, vitamin deficiencies, or use of medications known to impair mood or cognition;
(vi) Substance use: alcohol abuse or illicit substance dependence within the past 6 months;
(vii) Contraindications to neurostimulation: history of craniotomy, presence of implanted medical devices (e.g., pacemakers, deep brain stimulators), metallic splinters or prostheses, or personal/family history of epilepsy;
(viii) Pregnancy or lactation.

Patients will be included in the study only after providing written informed consent. All procedures will adhere to the principles of the Helsinki Declaration of 1964 and subsequent amendments. The study will be conducted by experienced professionals in a specialized environment to ensure safety and compliance with ethical standards.

Patients will discontinue participation in the study under the following circumstances: withdrawal of consent to participate in the study; voluntary abandonment of the study without formally withdrawing consent; death of the patient; occurrence of a serious adverse event that, in the clinical judgment of the investigators, significantly impairs the patient’s ability to provide valid data (e.g., acute cerebrovascular events); emergence of symptoms indicative of a hypomanic or manic episode. To promote participant retention and minimize loss to follow-up, participants will receive reminder calls before each follow-up visit. Flexible scheduling and telephone assessments will be offered when in-person visits are not possible.

Ethics and registration: the study was approved by the local Ethics Committee “Comitato Etico Catania 1” (Azienda Ospedaliero-Universitaria Policlinico “G. Rodolico–San Marco”, Catania, Italy) by Deliberation No. 1149 dated 24 May 2023. The trial is registered on ClinicalTrials.gov (NCT06714643; Unique Protocol ID 130/2022/PO; first posted 2024-12-03). All participants will provide written informed consent; all procedures comply with the Declaration of Helsinki and its amendments. 

### 2.2. Equipment

All interventions will be conducted at the Psychiatry Unit of the University Hospital Policlinico “G. Rodolico–San Marco” of Catania (Italy), under the supervision of a licensed mental health practitioner. The Brainstim low-intensity transcranial electrical stimulator (EMS, Integrated Solutions for Neuroscience, Bologna) will be used to deliver stimulation to the left DLPFC.

## 3. Procedure

### 3.1. Clinical and Psycho-Cognitive Assessment

After verifying the eligibility criteria and collecting demographic data, all participants will undergo a comprehensive baseline assessment (T0) designed to gather a wide range of clinical, neuropsychological, and physiological data. The clinical and neuropsychological evaluation will include the assessment of depressive symptoms using the HRSD-17 and a detailed examination of cognitive functioning through several standardized tests: MMSE, MoCA, FAB, and Stroop T. These tools will provide a thorough overview of both mood and cognitive status at the start of the study. In particular, the FAB is focused and very sensitive to executive functions, which are frequently affected in depression and often linked to DLPFC hypoactivity [57,58]. Regarding MoCA, although originally developed for screening mild cognitive impairment and dementia, it offers a broader assessment of multiple domains (e.g., attention, memory, visuospatial abilities, language, and executive function) that may also be affected in depression [59,60]. Together, these instruments provide a pragmatic and time-efficient approach to screen both executive deficits and global cognitive performance in our patient cohort. As such, their inclusion will allow correlation of cognitive changes with multidimensional outcomes.

### 3.2. Laboratory Assessment

In addition to these evaluations, a venous blood sample will be collected from each participant. These samples will be stored in 5 mL vacuum tubes containing anticoagulant and processed promptly. They will be centrifuged at 2000× g and maintained at 4 °C for 10 min within two hours of collection. The resulting serum will then be frozen at −70 °C for future analysis. Biomarkers known to play a role in depression, such as BDNF, NGF, VEGF, IGF1, Ang, Nrg1, IL-6, and TNF-α, will be measured using advanced techniques like enzyme-linked immunosorbent assay (ELISA), Western blotting, and immunoprecipitation kits.

### 3.3. Neurosonological Assessment

Cerebral hemodynamics will be assessed using transcranial Doppler ultrasonography (TCD), a widely used, non-invasive method to evaluate intracranial blood flow dynamics. Recordings will be obtained through a 2 MHz pulsed Doppler probe placed over standard acoustic windows. The middle cerebral arteries (MCA) will be insonated bilaterally through the transtemporal window, whereas the basilar artery (BA) will be evaluated via the transforaminal window. Depths of insonation will be adjusted (45–65 mm for MCA, 80–100 mm for BA) to optimize signal quality.

The following parameters will be recorded [61]:

(i) Peak systolic velocity (PSV): the maximal velocity during systole, reflecting arterial inflow;
(ii) End-diastolic velocity (EDV): the velocity during end-diastole, influenced by downstream resistance;
(iii) Mean blood flow velocity (MBFV): calculated as the time-averaged velocity over the cardiac cycle, representing global perfusion of the insonated vessel;
(iv) Pulsatility index (PI): calculated as (PSV—EDV)/MBFV, serving as a marker of cerebrovascular resistance and arterial compliance;
(v) Resistivity index (RI): calculated as (PSV—EDV)/PSV, also reflecting vascular resistance.

Measurements will be performed under resting conditions and repeated during a standardized 30 s breath-holding test (BHT) to assess cerebrovascular reactivity (CVR). The breath-holding index (BHI), defined as the relative increase in MBFV after BHT, will be derived as an indirect marker of vasomotor reserve [62,63].

The clinical relevance of these measures lies in their ability to detect subtle impairments in cerebral perfusion and vascular compliance, which are often linked to depressive syndromes with vascular burden. In particular, increased PI and RI have been associated with microvascular damage, reduced vasomotor reactivity, and cognitive impairment, especially in vascular depression [50,51,64]. By combining resting and reactive measurements, this protocol provides a comprehensive assessment of cerebrovascular physiology and its potential modulation by tDCS.

### 3.4. Neurophysiological Assessment

Cortical excitability will be assessed with single- and paired-pulse TMS using a figure-of-eight coil connected to a magnetic stimulator. The coil will be positioned tangentially to the scalp over the left primary motor cortex (M1), with the handle pointing backward and laterally at a 45° angle to the sagittal midline, to optimally induce posterior–anterior current flow in the underlying cortex [65]. The stimulation “hot spot” will be defined as the scalp position eliciting the largest and most consistent motor-evoked potentials (MEPs) in the contralateral first dorsal interosseous (FDI) muscle. Electromyographic activity will be recorded from surface electrodes placed on the FDI.

The following parameters will be obtained [65]:

(i) Resting motor threshold (rMT): defined as the minimum stimulus intensity required to evoke MEPs of ≥50 μV in at least 5 of 10 trials at rest, reflecting global corticospinal excitability.
(ii) MEP amplitude: an index of corticospinal tract integrity and responsiveness.
(iii) Central motor conduction time (CMCT): derived from the latency difference between spinal and cortical stimulation, reflecting conduction efficiency.
(iv) Contralateral silent period (cSP): measured during tonic muscle contraction and reflecting GABA-B-mediated intracortical inhibition.
(v) Ipsilateral silent period (iSP): elicited by suprathreshold stimulation of M1 during contraction of the homolateral FDI, serving as a marker of transcallosal inhibition.

Paired-pulse paradigms will assess short-interval intracortical inhibition (ICI), reflecting GABA-A receptor activity, and intracortical facilitation (ICF), associated with glutamatergic excitatory transmission. Conditioning stimuli will be set at 80% of rMT and test stimuli at suprathreshold intensity, with interstimulus intervals of 2–5 ms for ICI and 10–15 ms for ICF. These measures provide insight into the balance between excitatory and inhibitory circuits and allow the detection of neuroplastic changes induced by tDCS [41,66].

Overall, this multimodal TMS protocol integrates several validated indices of cortical excitability and inhibition, enabling exploration of the physiological correlates of depressive symptomatology and their modulation by tDCS.

### 3.5. Randomization and Blinding

Patients will be randomly assigned to one of two groups to ensure unbiased allocation: a test group, which will receive active tDCS, and a sham group, which will receive simulated tDCS. To minimize systematic differences between the groups, randomization will be conducted using specialized software (RStudio version 4.2.1, with the randomizeR and blockrand packages) that stratifies patients by age, sex, and residual symptoms. A reference document linking patient randomization codes to their names will be maintained by the principal investigator, ensuring secure record-keeping while maintaining confidentiality for other team members. Blinding procedures will be rigorously applied. Patients will remain unaware of their group assignment, as the administration process will appear identical for both the active and sham groups. To maintain assessor blinding, two distinct roles will be assigned: recruiters and treatment administrators, who will be aware of the group allocation and responsible for delivering the intervention; evaluators, separate from the recruiters and administrators, who will assess patient outcomes without participating in the stimulation sessions and will remain blinded to the treatment type. After randomization, patients will undergo a follow-up evaluation using the same protocol as the baseline assessment. Blinding will be preserved for patients throughout the entire study, ensuring that their knowledge of the treatment type does not influence the outcomes. Unblinding will be permissible only in exceptional cases where knowledge of the assigned intervention is essential for the clinical management of the participant (e.g., serious adverse event). The decision to unblind will be made by the principal investigator and documented in the trial records. The data analysis team and outcome assessors will remain blinded regardless of individual unblinding events.

### 3.6. Interventions and Study Duration

The protocol for both active tDCS and sham tDCS involves 15 sessions of excitatory tDCS distributed over three weeks (five sessions per week, Monday through Friday), conducted at the same time each day (approximately 9:30 a.m.), by the same operators, and under identical experimental conditions. Each session will last 30 min, during which the anode electrode will be placed over the left DLPFC and the cathode electrode over the right DLPFC, with a stimulation intensity of 2 mA.

Electrode placement will follow the international 10–20 EEG system. The anodal electrode will be positioned over F3 (corresponding to the left DLPFC), while the cathodal electrode will be positioned over F4 (right DLPFC). According to the classification of Nasseri et al. (2015) [67], this montage represents a bilateral frontocortical configuration, intended to increase excitability in the left DLPFC and decrease excitability in the right DLPFC. This interhemispheric modulation is hypothesized to restore functional asymmetry in prefrontal activity, a well-documented feature of MDD. In the sham procedure (sham tDCS), which functions as a placebo treatment, the electrodes will be positioned identically on the scalp. However, only an initial stimulation current lasting 15 s will be delivered, creating a brief sensation of current on the scalp to mimic the experience of active tDCS. Participants in the sham group will also hear similar sounds and experience stimuli resembling those in the active tDCS sessions.

To ensure blinding, all patients will remain unaware of the type of treatment they are receiving throughout the study. The study will span 24 months, including a clinical follow-up period of 9 months. Each patient will participate in the study for a total of 29 weeks, comprising three weeks of treatment followed by 26 weeks of follow-up. Given the relatively long duration of the study, independent personnel responsible for data monitoring will be considered. A plain-language summary of the results will be made available to interested participants upon request.

### 3.7. Sample Size and Statistical Analysis

The primary outcome of the study is to evaluate the difference in depressive symptom severity, as measured by the HDRS-17 scale, following the intervention. Assuming a normal distribution of HDRS-17 scores at the study endpoint, the scores of the two groups will be compared using an independent-samples Student’s *t*-test. The sample size calculation was based on the most recent data from the literature [68,69]. According to these findings, tDCS treatment in patients with unipolar depression is associated with a symptom reduction corresponding to a Hedges’ g effect size of 0.55. Using G*Power software version 3.1.9.6 for sample size estimation, with an alpha level of 0.05 and beta set at 0.20 (equivalent to a power of 0.80), the required sample size is 84 participants, with 42 allocated to each study arm. To account for a potential dropout rate of 20%, the study aims to recruit at least 105 participants. For missing data, appropriate statistical methods will be applied, utilizing the last recorded score if it was measured post-baseline. To address multiplicity in the biomarker panel, we will control the false discovery rate (FDR) at 5% using the Benjamini–Hochberg procedure. The adjustment will be applied within the biomarker family (*n* = 10; neurotrophic and inflammatory markers) for each pre-specified contrast of interest. We will report FDR-adjusted *p*-values (*q*-values) alongside effect sizes and 95% confidence intervals.

Specifically, the last observation carried forward (LOCF) technique will be employed. The analysis will follow a modified intention-to-treat (m-ITT) approach, including only those participants who have at least one post-baseline measurement for the primary outcome. Regardless of the data availability, participants will be analyzed according to their original randomization group. Any deviations from the statistical analysis plan will be thoroughly documented and reported as protocol deviations in the appendix of any publications arising from this study. Exploratory subgroup analyses may be conducted based on demographic or clinical variables (e.g., age, sex, baseline HDRS-17 score) to assess differential treatment response. Sensitivity analyses will be performed to test the robustness of findings, particularly in relation to protocol deviations and missing data handling. No interim analyses or formal stopping guidelines are planned for this study. Given the short duration of the intervention and the low-risk nature of the non-invasive procedure (tDCS), the trial will proceed as scheduled unless unexpected safety concerns arise. Any serious adverse events will be reviewed by the principal investigator, who holds the authority to suspend or terminate the trial if necessary. No data monitoring committee (DMC) is established for this trial.

This is a single-center clinical trial involving a non-invasive, low-risk intervention (tDCS), with a limited sample size and short treatment duration. All safety assessments and data integrity checks will be conducted internally by the research team. Given the minimal risk to participants, a formal DMC is not deemed necessary.

### 3.8. Table and Figure

Table 1 graphically represents when each evaluation will be performed at baseline, post-intervention, and follow-ups at 3 and 6 months, whereas Figure 1 shows the flow diagram of the study protocol.

## 4. Expected Results

This study represents a meaningful advancement as the first randomized controlled trial to investigate the relationship between the efficacy of tDCS in treating depressive symptoms and its effects on inflammation and neurotrophic factors. While certain biomarkers, such as BDNF and VEGF, have already been associated with depression and its treatment, the evidence for other markers remains inconsistent and requires further exploration. By addressing these gaps, this trial has the potential to not only confirm the role of established biomarkers but also to provide new insights into lesser-studied factors, ultimately contributing to a more comprehensive understanding of the biological changes induced by tDCS.

The findings from this trial are expected to have a significant impact on the future of depression treatment. By linking the clinical effects of tDCS—previously demonstrated in trials without a clear biological basis—to measurable changes in neurotrophic and inflammatory markers, this study will provide critical evidence to explain how tDCS may achieve its therapeutic benefits. Such insights will help clarify the neurobiological mechanisms at play, enabling a more informed application of tDCS in clinical settings and fostering its integration into routine practice.

Additionally: the results of this study will provide evidence-based data to support further research and innovation in the field of neuromodulation for depression. Understanding the short- and long-term biological effects of tDCS will not only refine its therapeutic role but also contribute to the development of future guidelines for depression treatment. This trial is uniquely positioned to bridge the gap between clinical outcomes and neurobiological mechanisms, paving the way for a deeper and more precise understanding of how tDCS can be used to improve the lives of patients suffering from this debilitating condition. The results of this clinical trial will be disseminated through publication in peer-reviewed scientific journals and presentation at national and international conferences. A summary of the study findings will be reported on ClinicalTrials.gov (accessed on 28 November 2024) (NCT06714643) upon study completion. Participants who express interest will be provided with a plain-language summary of the results. Relevant healthcare professionals and institutions involved in the care of study participants will also be informed of the findings.

## Figures and Tables

**Table 1:** Patients’ clinical, laboratory, and instrumental assessment timing.

Assessment Type	Baseline (T0)	Post-Treatment, 3 Weeks (T1)	3-Month Follow-Up (T2)	6-Month Follow-Up (T3)
Clinical evaluation	*✔*	*✔*	*✔*	*✔*
Cognitive assessment	*✔*	*✔*	*✔*	*✔*
Serum biomarker dosage	*✔*	*✔*	*✔*	*✔*
Cortical excitability by TMS	*✔*	*✔*	*✔*	*✔*
Cerebral hemodynamics by TCD	*✔*	*✔*	*✔*	*✔*

Legend T0 = baseline; T1 = immediately after the tDCS intervention (3 weeks); T2 = 3-month follow-up; T3 = 6-month follow-up; TMS = transcranial magnetic stimulation; TCD = transcranial Doppler sonography.

## Data Availability

Since the study is promoted by the Complex Operating Units of Neurology and Psychiatry at the University Hospital “G. Rodolico–San Marco,” the data controllers are the principal investigator and the co-principal investigator. In compliance with European Regulation 2016/679 (GDPR), the data and samples collected for the purposes of the study protocol will be retained only for the duration of the project. Upon completion, the patient’s data and samples will be destroyed unless they have been anonymized during the experimentation process, making it impossible to trace them back. If personal data are transferred to a third country or an international organization, all safeguards outlined in Article 46 of GDPR 679/2016 regarding such transfers will be implemented. Participants in the clinical trial may, at any time, appeal to the Data Protection Authority to safeguard their rights (Supplementary Materials).

## Supplementary Materials

The following supporting information can be downloaded at: https://www.mdpi.com/article/10.3390/mps8050117/s1, SPIRIT 2025 checklist of items to address in a randomized trial protocol [70].

## Abbreviations

The following abbreviations are used in this manuscript:

tDCS	transcranial direct current stimulation
MDD	major depressive disorder
NVU	neurovascular unit
DLPFC	dorsolateral prefrontal cortex
SSRIs	serotonin reuptake inhibitors
SNRIs	serotonin and norepinephrine reuptake inhibitors
HPA	hypothalamic–pituitary–adrenal axis
VEGF	vascular endothelial growth factor
IL-6	interleukin 6
TNF-α	tumor necrosis factor α
BDNF	brain-derived neurotrophic factor
NSCs	neural stem cells
NTs	neurotrophic factors
NGF	nerve growth factor
IGF-1	insulin-like growth factor 1
HIF-1 α	hypoxia-inducible factor-1 α
NRG1	neuregulin-1
NMDA	N-methyl-D-aspartate
sgACC	subgenual anterior cingulate cortex
fMRI	functional magnetic resonance
CRP	C-reactive protein
TrkB	tyrosine protein kinase B
MoCA	Montreal Cognitive Assessment
FAB	Frontal Assessment Battery
rMT	resting motor threshold
